# Carbohydrate Mouth Rinse Decreases Time to Complete a Simulated Cycling Time Trial

**DOI:** 10.3389/fnut.2019.00065

**Published:** 2019-05-15

**Authors:** Gabriel Baltazar-Martins, Juan Del Coso

**Affiliations:** ^1^Faculty of Sports Science, European University of Madrid, Madrid, Spain; ^2^Exercise Physiology Laboratory, Sport Science Institute, Camilo José Cela University, Madrid, Spain

**Keywords:** mouth rinse, carbohydrate, cycling performance, power output, training low

## Abstract

Rinsing carbohydrate solutions in the mouth can produce positive effects on the central nervous system via mouth/tongue receptors, ultimately increasing cycling performance. However, previous investigations on this topic have used complex carbohydrate solutions and time trials on a cyclergometer to complete a set amount of work. The purpose of the present study was to examine the effects of carbohydrate mouth rinsing on physical performance by using a commercially available drink during a cycling time trial with varying slopes. In a double-blind, placebo-controlled and randomized manner, 16 well-trained cyclists (37.6 ± 3.5 years; 76.9 ± 7.9 kg) performed two simulated cycling time trial (25.3 km) with their own bikes on a 3D virtual trainer. In one occasion, participants mouth-rinsed a 6.4% carbohydrate mixed solution for 5 s each 12.5% of total completion of the trial; in other occasion participants rinsed with a taste-matched placebo with 0.0% of carbohydrate. During the trials, participants were instructed to perform as fast as possible at a self-chosen pace while time, cycling power output and ratings of perceived exertion were obtained during the trials. When compared to the placebo, carbohydrate mouth rinse decreased the time employed to complete the distance (2,960 ± 412 vs. 2,888 ± 396 s; *P* = 0.04, respectively), while it increased overall cycling power (222 ± 51 vs. 231 ± 46 w, *P* = 0.04) and cycling power during the climbing sections (238 ± 46 vs. 248 ± 47 w, *P* = 0.03). Carbohydrate mouth rinse also increased the rating of perceived exertion at the end of the trial (18.3 ± 1.7 vs. 18.9 ± 1.1 arbitrary units, *P* = 0.04). In summary, mouth rinsing with a commercially available carbohydrate drink might be considered as an effective strategy to increase physical performance during cycling time trials. However, due to the performance downsides of breaking the aero-position or interrupting the breathing pattern for rising during a time trial, carbohydrate mouth rinse protocols might be more suitable for high-intensity training sessions, particularly those sessions intentionally performed with low carbohydrate intake.

## Introduction

In 2004, well-trained endurance cyclists were infused with either glucose (1 g/min) or saline (placebo) while they completed as quickly as possible an ~1 h cycling performance test (1). In this study, the infusion of glucose did not produce any ergogenic effect over saline infusion despite the infusion of glucose increased availability of plasma glucose and increased glucose uptake into the skeletal muscle. This seminal investigation leaded to question that the ergogenic effects of carbohydrate ingestion on endurance performance, established 20 years before (2–4), were somewhat related to the exposure of the oral cavity to carbohydrate. For this reason, Carter et al. (5) subsequently investigated the effect of rinsing with a 6.4% carbohydrate-based solution (without ingesting the solution), in comparison to water mouth rinse, on the same ~1 h cycling performance test. In this latter investigation, carbohydrate mouth rinse promoted an ergogenic effect on endurance cycling performance that was related to an increase in central drive or motivation because it was unrelated to any metabolic mechanism.

These investigations were pioneer because they led to the notion that rinsing nutrient solutions in the mouth during exercise could possibly exert an effect on the central nervous system via mouth/tongue receptors, which could promote an improved sense of well-being and a decreased rate of perception of effort (6, 7). Posterior studies have confirmed that these oral receptors could directly stimulate reward centers in the brain, leading to a positive “central drive” that would ultimately improve endurance exercise performance even in situations in which muscle and hepatic glycogen stores are not depleted (6, 8–12).

Carbohydrate mouth rinsing can be defined as flushing a carbohydrate-based drink around the oral cavity for certain time, followed by the subsequent expulsion of fluid (13). Recently, plenty of research associates carbohydrate mouth rinse to positive effects in athletic performance, not only in endurance activities but also with a growing interest in high intensity sprint-based activities (14–23). Recently, Brietzke et al. (7) have reviewed the effects of carbohydrate mouth rinse on cycling time trial performance and they have concluded that this technique is effective in increasing cycling power output, although the magnitude of the effect was cataloged as “small.” Despite the overall positive effect of carbohydrate mouth rinse on cycling performance, all studies reporting no beneficial of carbohydrate mouth rinse on cycling performance tested participants in a fed state (24–28). Although it seems that carbohydrate mouth rinse improved performance to a greater extent in a fasted compared with a fed state, the use of fasting prior to exercise might not be an optimal choice to maximize cycling performance. Therefore, the combination of fasting and carbohydrate mouth rinse might be a strategy more suitable to training sessions, especially for those sessions intentionally performed with low carbohydrate intake. However, the usefulness of carbohydrate mouth rinse might be limited in a competitive context where athletes usually perform a carbohydrate loading in the hours previous to the competition to increase liver and muscle glycogen stores.

In addition, the effect of carbohydrate mouth rinse on cycling performance has been investigated by using protocols on a cyclergometer consisting of completing an amount of work as quickly as possible or exercise at a fixed workload until exhaustion (7), applicable to real cycling time trials on flat courses. Although these types of performance test have validity and reliability, they are based on the maintaining of a relatively constant power output during the trial and thus, they lack the change in intensity and slope of mountain courses. In addition, previous studies on carbohydrate mouth rinse have used beverages with maltodextrin, glucose, and sucrose (7), while most of these solutions are not available in the market. Thus, the aim of the present study was to determine the effects of carbohydrate mouth rinse, using a commercially available drink, on cycling performance. We used an ecologically valid context to assess cycling performance that included a pre-competition meal and the simulation of a 25.3-km time trial with varying slopes. We hypothesized that carbohydrate mouth rinse would enhance cycling time-trial performance in well-trained cyclists when compared to mouth-rinsing a placebo solution.

## Materials and Methods

### Participants

Sixteen male and well-trained cyclists volunteered to participate in this study (age = 37.6 ± 3.5 years; body mass = 76.9 ± 7.9 kg; cycling experience > 7 years). Participants trained > 4 days per week, > 60 min of training duration per day and they competed > 5 times per year in the last 5 years. Prior to the onset of the experiment, all the participants underwent a pre-participation screening that included a medical and training history. All the participants were non-smokers, who had no previous history of cardiopulmonary diseases or musculoskeletal injuries in the previous 3 months. The participants were encouraged to avoid medications or nutritional supplements for the duration of the study. One week before the onset of the study, the participants were fully informed of the experimental procedures and the risks and discomforts associated with the research and gave their informed written consent to participate in the investigation. The study was approved by the University Research Ethics Committee and has been performed in accordance with the ethical standards as laid down in the 1964 Declaration of Helsinki and its later amendments or comparable ethical standards.

### Study Design

A double-blind, placebo-controlled, randomized, and cross-over experimental design was used in this study. Each participant took part in 2 identical protocols and thus acted as his own control: in one protocol, participants performed a simulated cycling time trial in a 3D virtual training simulator and rinsed their mouths with a carbohydrate beverage containing 6.4% carbohydrate concentration (Carrefour® sport drink, France); in another protocol, participants performed the same cycling trial but rinsed their mouths with a tasted-matched placebo beverage with 0.0% carbohydrate concentration (Carrefour®, sport drink Zero, Spain). The beverage for the carbohydrate mouth rinse protocol contained a mix of different types of carbohydrate (sucrose, glucose, and fructose) while the beverage for the placebo mouth rinse protocol was identical in appearance and taste but contained artificial sweeteners (sucralose and acesulfame-K). The full list of ingredients of each drink is described on Table 1. The use of a drink with artificial sweeteners is a valid beverage to create a placebo mouth rinse protocol as this type of drink does not activate the same cerebral structures involved in the reward system (8, 29) that a carbohydrate drink has been shown to activate. The participants did not report being able to distinguish a difference between the carbohydrate and placebo solutions. The order of the mouth rinse protocols was randomized, and they were separated by 7 days to allow for complete recovery. An alphanumeric code was assigned to each trial by a person who was independent of the investigation to blind participants and investigators to the drink tested.

**Table 1:** List of ingredients of the carbohydrate drink and the placebo drink.

**Carbohydrate drink**	**Placebo drink**
Natural mineral water (83%), orange juice (10%), sugar, glucose, and fructose syrup, acidifier: citric acid, stabilizers: locust bean gum and pectin, antioxidant: ascorbic acid, coloring: carotenes, natural orange flavoring and other natural flavorings.	Natural mineral water 96%, acidulant: citric acid, antioxidant: ascorbic acid, natural orange flavor with other natural aromas, sweeteners: acesulfame and sucralose, stabilizers: pectin and gum, coloring: carotenes.

### Standardizations

One week before the onset of the experiment, participants underwent a routine medical screening to ensure that they were in good health and suitable for the experiment. On this day, participants completed a familiarization trial that replicated all the settings included in the experimental trials. In the familiarization trial, the mouth rinse protocol was performed with tap water and participants tested both 5-s and 10-s mouth rinses to select the rinse duration that less interfered with the trial. For the 48 h before the onset of the experiments, participants refrained from all sources of dietary caffeine, alcohol, and stimulants. Participants were also encouraged to maintain their training routines and to keep a stable fitness state during the whole experiment, although strenuous exercise was avoided 48 h before the onset of the experimental trials to taper for the simulated time trial. On the 24 h preceding the first experimental trial, subjects recorded their dietary and fluid intake by photographs and they were asked to replicate this diet pattern on the second trial. Participants were encouraged to consume their habitual pre-competition meal, with at least 3 g/kg of body mass of carbohydrate, 3 h before the start of testing and replicated this before the second experimental trial. Adherence to these standardizations was checked verbally and visually before each trial. Environmental temperature and humidity were kept constant in all experimental trials (21.3 ± 0.3°C air temperature and 50 ± 10% relative humidity). Standardized encouragement and feedback were given to the participants in all trials by the same researcher who was blinded to the treatments. Participants were not allowed to listen to music during the experiments. The seat and handlebar positions on the bikes and tire pressure were obtained in the familiarization trials and replicated for each participant in all trials. Tire pressure was checked with a digital bike tire gauge before the experimental trials.

### Experimental Protocol

**Figure 1:**
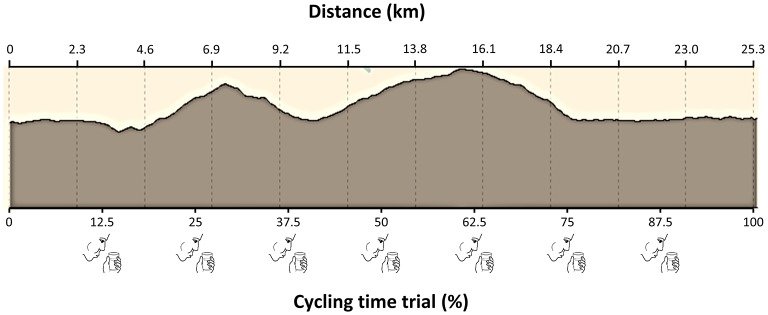
Profile of the cycling time trial (25.3 km). Participants mouth-rinsed with a 6.4% carbohydrate beverage or a tasted-matched non-caloric placebo beverage during the trial.

Both experimental trials (i.e., carbohydrate vs. placebo mouth rinse protocols) took place at the same time of the day (from 16:00 to 20:00). Participants arrived at the laboratory, emptied their bladders and their nude body mass measured afterwards (B-418, Tanita, Japan). Participants then dressed in a T-shirt, and shorts and performed a 10-min standardized warm-up on their own road bikes. After this, participants performed a simulated time trial consisting of completing a 25.3-km course as fast as possible. The trial was performed on a 3D cycling simulator (Smart Pro®, Bkool, Spain) which has an embedded adaptation mechanism that induces more or less resistance on the bike according to the slope shown in a screen, providing a similar feeling of pedaling on cycling route (30). This means that resistance was constantly varying depending on slopes and instant cycling velocity and participants had to adjust their cycling power output by changing pedaling cadency and shifting gears, as it happens in a real cycling route. Accordingly, in the downhill sections of the virtual route, the simulator decreased the resistance to pedaling in a similar manner to a real downhill descent. A pilot study with 10 amateur cyclists indicated that the coefficient of variation for the time employed to complete a cycling time trial was ~2% when they performed twice the same 20-km course with the cycling simulator. The course for the cycling trial was intentionally chosen because it has several variations on the slope of the track, as it happens during a mountain time trial (Figure 1). Because we intended to test the effects of carbohydrate mouth rinse during a mountain time trial, we chose a course with a climbing distance of ~15% of the trial distance, as well as a total elevation gain > 400 m (specifically, 432 m of elevation gain for the selected course). Participants were encouraged to complete the trial as fast and possible while exercise intensity (i.e., power output) was individually modulated by changes in slope, pedaling frequency, and shifting gears. In the last 500 meters of cycling time trial, participants were instructed to perform an all-out sprint to obtain their peak power output. During the time trial, participants had visual access to the route profile and current slope in a computer screen, so they could anticipate and adapt gear shifting. However, participants were blinded to elapsed time, speed, power output, and cadence information on screen and they obtained information about the time employed in each trial after the experiments were concluded. During the trial, once the 12.5% of the total cycling distance was completed, participants rinsed in their mouths 25 mL of the experimental beverages, following the protocol by Carter et al. (5). The solution mouth rinse was repeated each 12.5% of the total cycling distance for a total 7 mouth rinses in each experimental trial. Each mouth rinse lasted for 5 s and then participants were asked to spit the content to a glass provided by the investigator. Although several studies used 10-s rinse protocols (7), we selected a 5-s rinse time because participants chose this rinsing protocol, over the 10-s protocol, during the familiarization trial −75% of participants (12 out of 16) considered that 10-s of mouth rinsing negatively affected their breathing pattern during cycling. In addition, previous research has found positive results with 5-s carbohydrate mouth rinse protocols on cycling performance (9, 10). During the trials, participants were not allowed to consume any drink. Along with each mouth rinse, partial ratings of perceived exertion, measured with the 6-to-20 point Borg Scale, were registered (31). The rating of perceived exertion was also obtained at the end of the time trial. Once the cycling test was concluded, body mass was registered with the same apparatus and the experiment was finished. In a different day, total time employed to complete the 25.3-km, mean power output recorded by the virtual training, power output during the climbing sections (> 4% of slope) and peak power output during the sprint, were obtained by an investigator blinded to the treatments.

### Statistical Analysis

Data were collected as previously indicated and the results of each test were subsequently blindly introduced into the statistical package SPSS v 20.0 for analysis. Normality was tested for each variable with the Shapiro-Wilk test. All the variables included in this investigation presented a normal distribution (*P* > 0.05) and parametric statistics were used to determine the ergogenicity of carbohydrate mouth rinse. Differences between carbohydrate vs. placebo mouth-rinsing protocols in cycling time and power output were determined by paired samples *T*-tests. The differences in the ratings of perceived exertion where were determined by two-way analysis of variance (mouth-rinsing protocol × cycling section) with repeated measures. After a significant *F*-test (Geisser-Greenhouse correction for the assumption of sphericity), differences between means were identified using Tukey’s HSD *post-hoc*. The significance level was set at *P* < 0.05. The effect size was also calculated in all pairwise comparisons to allow a magnitude-based inference approach (32). Specifically, the effect-size statistic ± 90% confidence intervals (CI) was used on log transformed data to reduce bias due to non-uniformity of error. Effect sizes were interpreted according to the following ranges: <0.2, trivial; 0.2–0.6, small; 0.6–1.2, moderate; 1.2–2.0, large; 2.0–4.0, very large and; >4.0, extremely large (32).

## Results

In the carbohydrate mouth rinse trial, body mass changed from 76.9 ± 7.9 to 75.9 ± 7.9 kg (*P* < 0.05, *t* = 13.6), which represents a body mass decrease of 1.40 ± 0.42%. In the placebo trial, body mass changed from 76.9 ± 7.9 to 75.8 ± 7.9 kg (*P* < 0.05, *t* = 15.1), which constitutes a body mass decrease of 1.42 ± 0.41%. However, there were no statistical differences in body mass decrease between the two experimental trials (*P* = 0.58, *t* = −0.6; ES = 0.1 [−0.1 to −0.3]).

**Figure 2:**
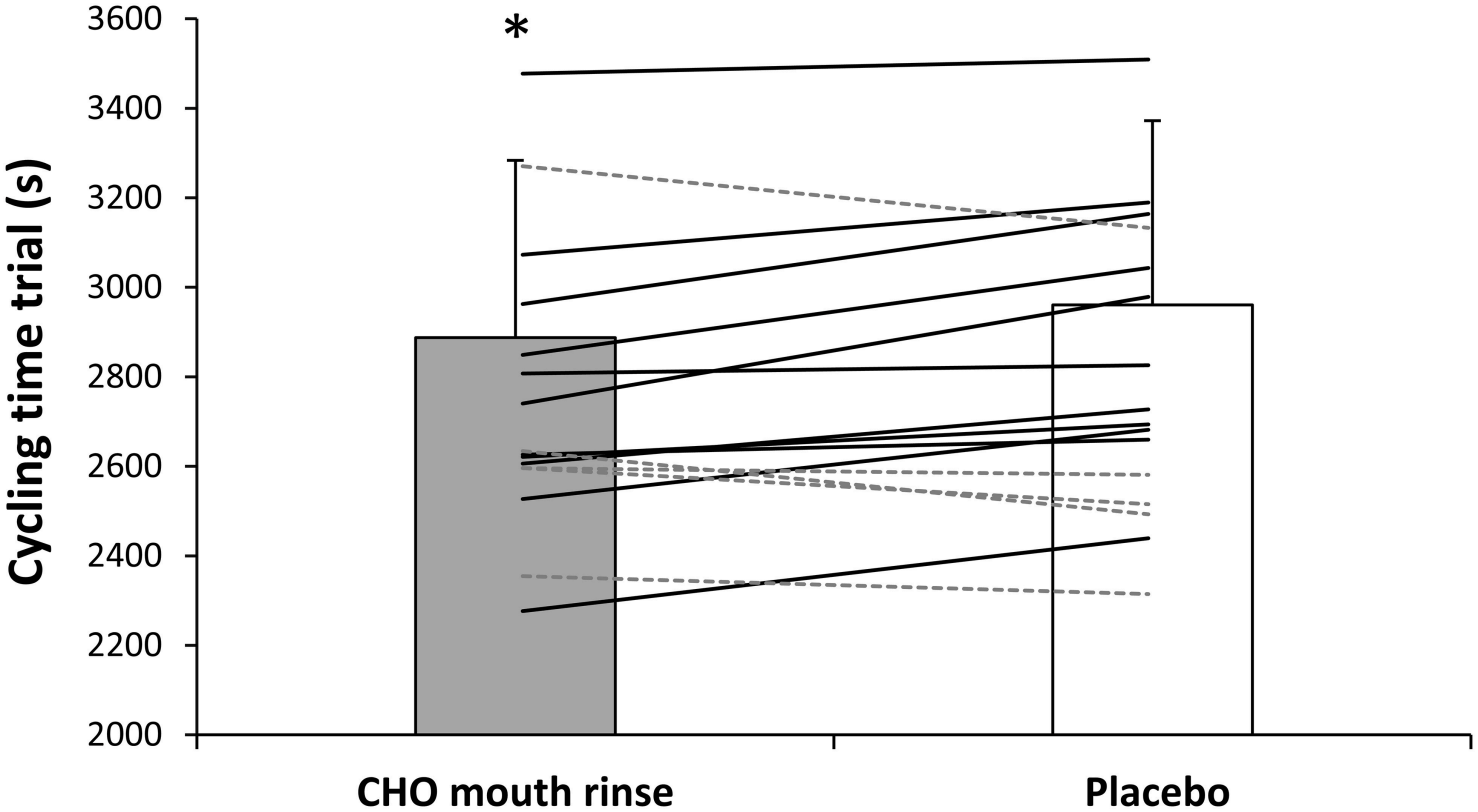
Time employed to complete the cycling time trial with carbohydrate mouth rinsing or with placebo mouth rinsing. Solid lines represent participants with lower times in the carbohydrate mouth rinsing protocol vs. placebo. Dashed lines represent participants with higher times in the carbohydrate mouth rinsing protocol vs. placebo. *Different from placebo at *P* < 0.05.

**Figure 3:**
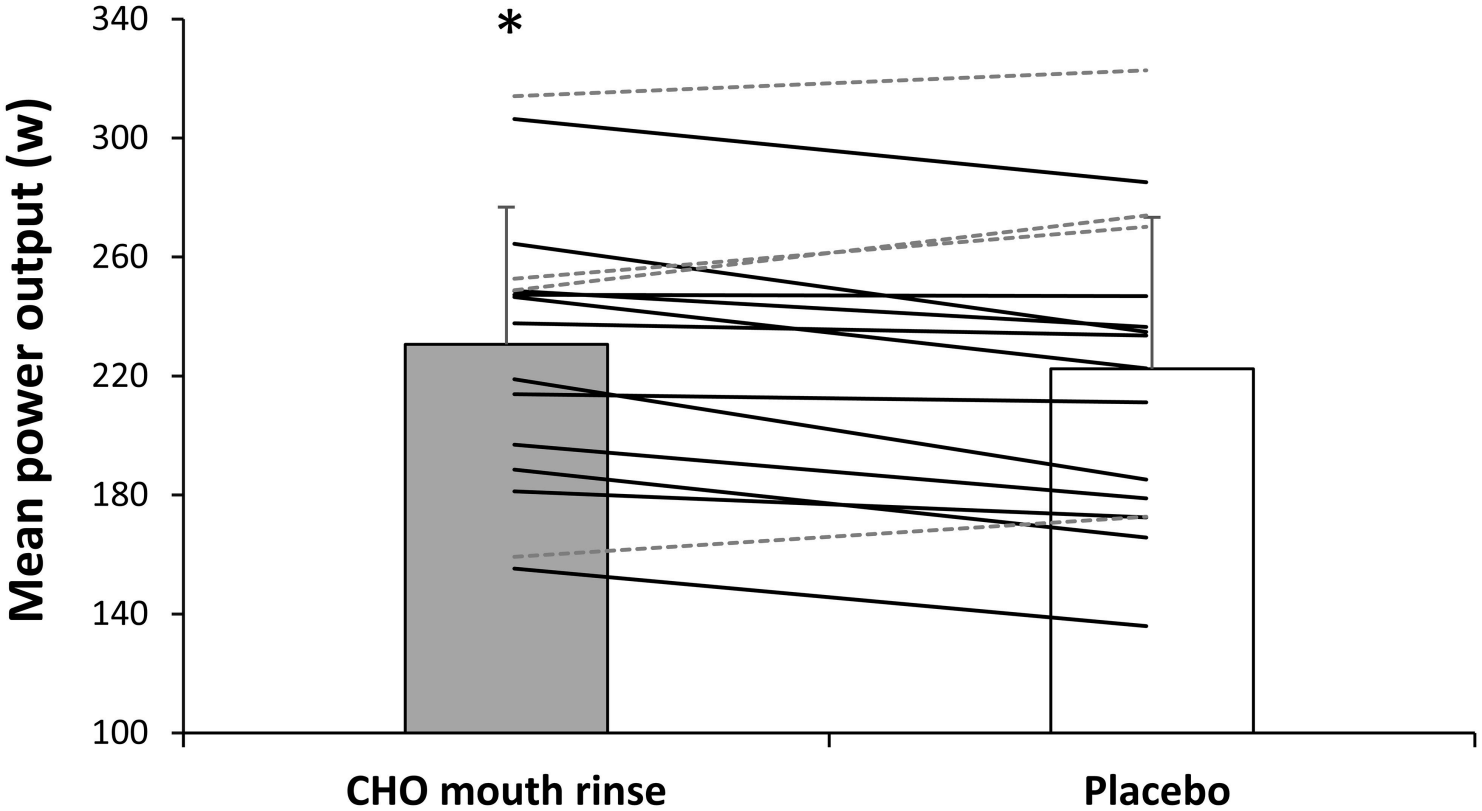
Mean power output during the cycling time trial with carbohydrate mouth rinsing or with placebo mouth rinsing. Solid lines represent participants with higher power output in the carbohydrate mouth rinsing protocol vs. placebo. Dashed lines represent participants with lower power output in the carbohydrate mouth rinsing protocol vs. placebo. *Different from placebo at *P* < 0.05.

**Figure 4:**
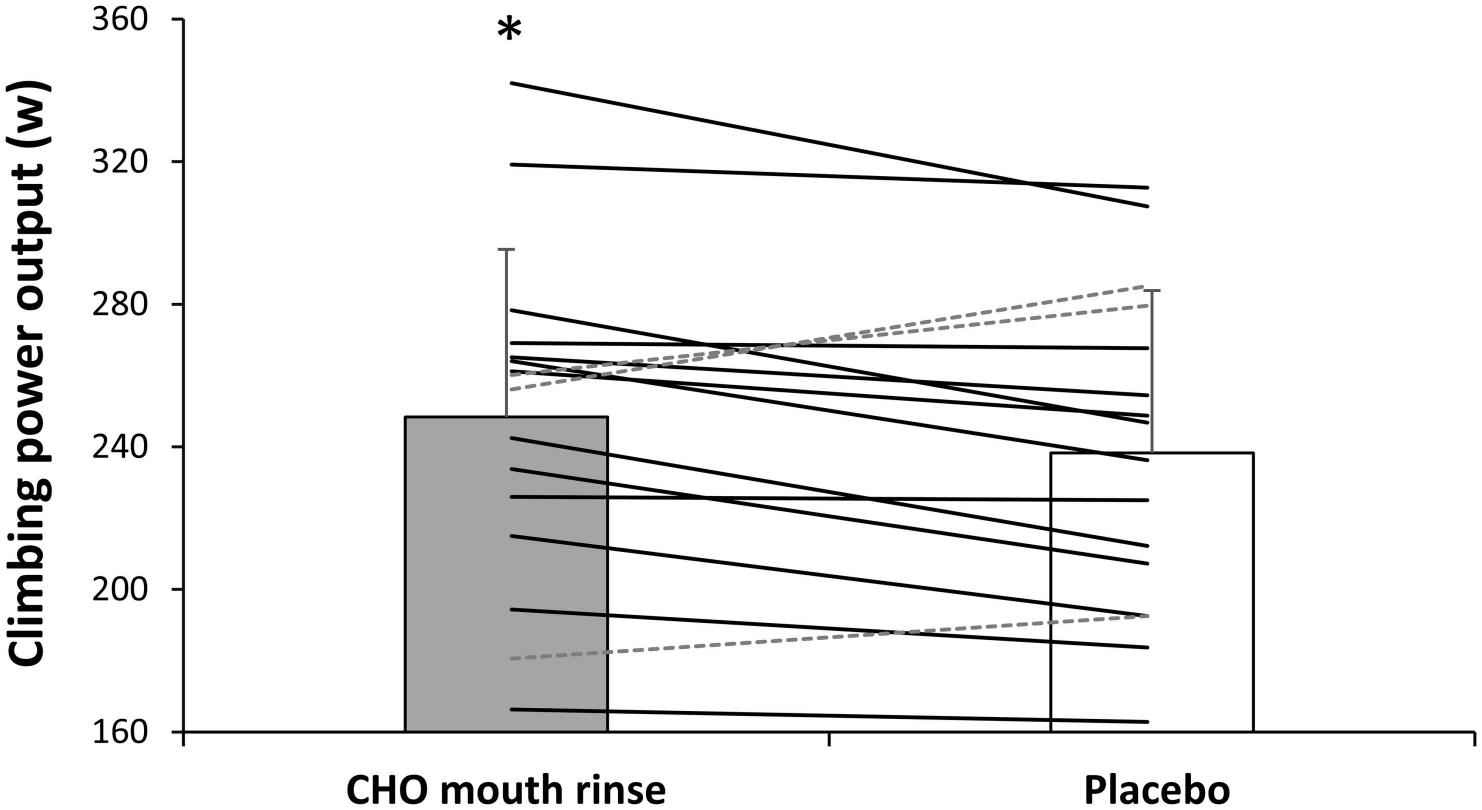
Cycling power output during the climbing sections of the time trial with carbohydrate mouth rinsing or with placebo mouth rinsing. Solid lines represent participants with higher power output in the carbohydrate mouth rinsing protocol vs. placebo. Dashed lines represent participants with lower power output in the carbohydrate mouth rinsing protocol vs. placebo. *Different from placebo at *P* < 0.05.

In the carbohydrate mouth rinse trial, the time employed to complete the cycling trial was reduced by 2.5 ± 5.1% respect to the placebo (*P* = 0.04, *t* = −3.8; ES = 0.2 [0.0–0.4]; Figure 1). Out of the 16 participants, 11 participants reduced their times to complete the trial with the rinsing of carbohydrate (Figure 1). Likewise, mean cycling power output was superior with carbohydrate mouth rinse vs. placebo (231 ± 46 and 222 ± 51 w, respectively) by 3.7 ± 9.4% (*P* = 0.04, *t* = 3.6; ES = 0.2 [0.0–0.4]; Figure 2). Out of the 16 participants, 12 participants increased their cycling power output with the carbohydrate mouth rinse (Figure 2). When considering only the climbing sections of the trial, the use of carbohydrate mouth rinse was also effective in increasing cycling power output (248 ± 47 and 238 ± 46 w,) by 4.2 ± 3.1% (*P* = 0.03, *t* = 4.2; ES = 0.2 [0.0–0.4]; Figure 3). Out of the 16 participants, 13 participants increased their cycling power during the climbing sections with the carbohydrate mouth rinse trial (Figure 3). The mouth rinse protocol also increased cycling power output in 12 out of the 16 participants during the non-hill sections of the time trial, in comparison to rinsing the placebo drink (227 ± 45 and 219 ± 52 w, respectively; *P* = 0.05, *t* = 3.5; ES = 0.2 [0.0–0.4]). In contrast, peak cycling power was similar in the carbohydrate mount rinse and placebo trials (731 ± 272 and 699 ± 234 w, respectively; *P* = 0.46, *t* = 0.7; ES = 0.1 [−0.3 to −0.2]). The rating of perceived exertion was similar during the two experiments (Figure 4) but the carbohydrate mouth rinse increased the rating of perceived exertion at the end of the trial (*P* = 0.04, *t* = 3.8; ES = 0.3 [0.0–0.6]).

## Discussion

**Figure 5:**
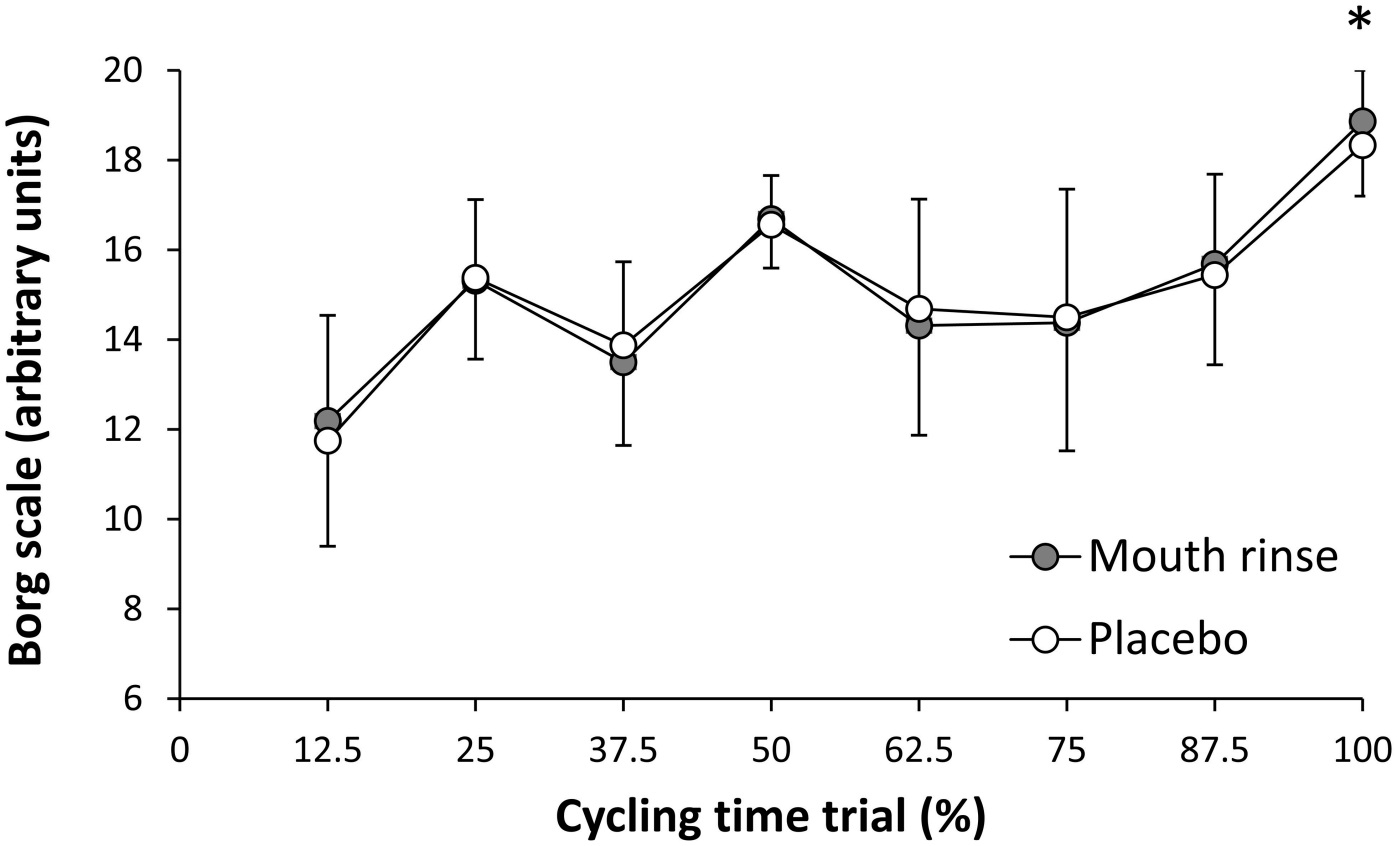
Rating of perceived exertion, measured with the 6-20-point Borg scale, during the cycling time trial with carbohydrate mouth rinsing or with placebo mouth rinsing. *Different from placebo at *P* < 0.05.

The aim of the present study was to determine the effects of carbohydrate mouth rinse on cycling performance by using an ecologically valid context that included a pre-competition meal (e.g., fed state) and the simulation of a 25.3-km time trial with varying slopes that simulates the profile of some time trial stages. To accomplish this, the cyclists performed the time trial on their own bikes by using a cycling trainer that simulates the changes in slope of a cycling profile while the trials were carried out indoors were ambient conditions were kept constant. In this controlled and applicable context, and, in comparison to the placebo mouth-rinsing protocol, participants took less time to complete the cycling distance when they rinsed the carbohydrate-based solution because they were able to maintain a higher mean cycling power during the trial. The protocol of carbohydrate mouth rinse was effective to increase cycling power during both climbing and more flattish sections. Interestingly, participants indicated a higher rating of perceived exertion after the end of the trial with carbohydrate mouth rinse (Figure 5), likely as a result of the higher exercise intensity maintained during the whole test. Thus, these results suggest that mouth rinsing with a commercially available carbohydrate solution might be considered as an effective strategy to increase cycling performance. However, since time trials are cycling competitions that typically last < 1 h, in which proper bike positioning is one of the most critical factors for performance, breaking the cycling position to perform a carbohydrate mouth rinse may not be completely applicable to such context. Instead, such a strategy will probably be most applicable in the training context while performing high-intensity exercise sessions in the fasted state or while aiming to generate an energy deficit. During training sessions, carbohydrate mouth rinse may allow a better maintenance of exercise intensity despite reduced carbohydrate availability.

The main outcomes of this investigation coincides with the recent review and meta-analysis by Brietzke et al. (7) on the effects of carbohydrate mouth rinse on cycling time trial performance. These authors systematically reviewed 16 randomized and placebo-controlled trials that assessed carbohydrate mouth rinse effects on mean power output and time to complete the trial and they concluded that mouth rinse improved mean power output with a standardized mean difference of 0.25 (95% CI 0.04–0.46). In the present investigation, the carbohydrate mouth rinse protocol also produced a significant increase in mean power output with the standardized mean difference was very comparable (0.2, 90% CI 0.0–0.4). However, the effect of rinsing carbohydrate during exercise on the time to complete the cycling trials did not reach statistical significance in the meta-analysis by Brietzke et al. (7) with a standardized mean difference of 0.13 (95% CI 0.10–0.36) while this effect was present in the current investigation (0.2, 90% CI 0.0–0.4). Taken together, this information suggests that carbohydrate mouth rinsing might have the capacity to increase cycling performance in time trials although the magnitude of the effect can be cataloged as small. Still, the applicability of carbohydrate mouth rinse during time trials remains questionable.

A methodological concern in the meta-analysis by Brietzke et al. (7) is the combination of experiments in which participants performed the time trial in a fasted/fed state. Interestingly, these authors indicated in their manuscript that all studies reporting no benefits of carbohydrate mouth rinsing on cycling performance standardized the pre-exercise meal (24–27), which might have reduced the overall magnitude of the carbohydrate mouth rinse effect found in their meta-analysis. It is interesting to note that cortical responses to the presence of carbohydrates in the mouth (in the form of sucrose) activates more brain regions in the fasted state compared with the fed state (29). Based on these studies, it has been postulated that carbohydrate mouth rinse improves performance to a greater extent in a fasted compared with a fed state because there may be greater activation of cerebral structures involved in the reward system in the presence of hunger (15). The current investigation challenges this postulation because carbohydrate mouth rinsing was effective to increase cycling performance in cyclists that performed a tapering protocol that included a reduction of exercise intensity and volume and nutrition strategies such a pre-time trial meal rich in carbohydrates. Although the reasons for the increased performance with the rinse of carbohydrate in our fed participants is no evident from our data, more research is needed to clearly stablish whether feeding/fasting is a definitive fact to discard/obtain the benefits of carbohydrate mouth rinse during exercise.

Similarly to the current study, several investigations compared carbohydrate mouth rinse to an artificially sweetened placebo (6, 15, 33). Other studies used maltodextrin as the carbohydrate solution and thus, they used water rinse as the control situation because the tasteless and colorless nature of maltodextrins (5, 18, 24, 34). Chambers et al. (6) studied the effect of rinsing with glucose and maltodextrin beverages and observed that both carbohydrate-based solutions activated areas of the brain involved in reward center in the brain. Based on the performance results of these investigations, it seems that several sources of carbohydrate can be used to rinse during exercise in order to provide a potential increase in performance (35), although a tasted-matched placebo should be used when experimenting the ergogenic effects of carbohydrate mouth rinse.

Sinclair et al. (18) reported that cycling performance was improved by doubling the duration (from 5 to 10 s) of the carbohydrate mouth rinse protocol, although their results were recently contradicted by Tomko (36) who found no dose-response effect when increasing the rinse duration from 5 to 15 s. Although more evidence is needed to ascertain the optimal rinsing time, it has been suggested that increasing rinsing duration may interfere with participants’ breathing patterns during high intensity exercise (34), as we found in our familiarization trial. Based on the information available so far, carbohydrate mouth rinsing for 5 s, each 5–10 min of exercise, allows for a significant contact between oral cavity and a carbohydrate source. This time seems enough to produce the activation of buccal/tongue receptors that can induce motor signaling, ultimately leading to improved performance during high-intensity endurance exercise. Similarly, increasing the carbohydrate concentration of the rinsed solution from 7 to 14% resulted in no further performance improvement (37). Thus, the use of 5-s rinsing protocols with standard commercially available sport drinks -which have a carbohydrate concentration between 6 and 8% (38)- might be the best manner of obtaining the benefits of carbohydrate mouth rinse during cycling.

One of the main novelties of this investigation is the use of a simulated time trial with continuous changes of slope (and thus exercise intensity) by means of a virtual trainer, where participants performed the trial on their own bikes. This protocol constitutes a novelty because previous investigations used exercise testing on cyclergometers and consisted of completing an amount of work as quickly as possible or exercise at a fixed workload until exhaustion, in which exercise intensity was kept relatively constant. Beyond removing the negative effect of adaptation to the equipment/cyclergometer, the current analysis provides a scenario that a course of varying slopes or hard training session, to determine the effects of carbohydrate mouth rinse on cycling performance. Lane et al. (15) used a similar protocol that included an indoor trainer and the use of subjects’ own bikes while they also find positive effects of carbohydrate mouth rinse. These data suggest that carbohydrate mouth rinse might be an effective strategy to increase cycling performance, as it has been previously found (5).

Despite the positive outcomes of carbohydrate mouth rinsing found in this investigation, the results of this investigation should be translated to real cycling competitions with caution. In a real cycling time trial, maintaining the cycling position on the bike is a key factor for performance. For this reason, removing the hands of the handlebar to reach the rehydration bottle from the cage on the frame might offset the performance benefits of carbohydrate mouth rinsing due to the loss of aerodynamics. In addition, it is possible that carbohydrate mouth rinsing is more useful to mountain bike or cyclocross competitions where cycling position is not as important to overall performance. While it seems impractical to interrupt breathing and/or removing the hands of the handle bar in a real competition scenario, giving the case that a cyclist might experience gastrointestinal distress with carbohydrate ingestion (39), this strategy might be only valid for those cyclists who are prone or are already feeling gastrointestinal distress during a training session or a race event. All this information, taken together, suggests that the performance benefits of carbohydrate mouth rinse should be balanced with the negative effects of this protocol on cycling position (especially aero position during time trials) and on the respiratory pattern during high-intensity exercise. The utility of carbohydrate mount rinse in a real cycling competition should be made individually in terms of type of competition, likelihood of suffering gastrointestinal distress, and after a careful familiarization period.

It is worth considering a number of limitations to the current study. First, the study sample was composed of amateur, although well-trained cyclists, that performed a cycling time trial of 25.3 km in ~48 min of duration. Thus, the application of these results to professional cyclists in longer races/competitions should not be generalized. To this respect, it is fundamental to verify the utility of carbohydrate mouth rinse protocol in the specific context of elite athletes, and to study whether the conditions of the cycling event (e.g., distance and elevation gain, conditions of the road, etc.) justify such approach. Second, although diet and exercise were standardized before the experimental trials, we did not obtain blood or muscle samples to assess the level of serum glucose, insulin or muscle glycogen stores. Third, the current study did not utilize a “no rinse” control situation, as suggested by Gam et al. (34), and we were unable to determine whether mouth rinsing *per se* during exercise was detrimental/beneficial for cycling performance. Fourth, we used a 3D cycling simulator with a coefficient of variation of ~2% for the measurement of the time employed to complete a cycling trial, which can contribute to the inter and intraindividual variability found in this investigation. Last, because a final sprint is common in many races, we set a 500 m all-out sprint at the end of the simulated time trial. However, this final sprint was not preceded of a mouth rinse, while the previous rinse was produced after the completion of 87.5% of the event (3.1 km before the finish line). It might be of interest in future studies to investigate if a carbohydrate mouth rinse has some effect in the capacity of performing a final sprint at the end of an exhausting trial when the rinsing protocol is performed just before the engagement in the sprint action. Despite all these limitations, the authors of this study consider that the current investigation adds some light to the literature on carbohydrate mouth rinse, particularly in relation to the use of a more ecologically valid context.

In summary, mouth rinsing with a 6.4% carbohydrate solution might be considered as an effective strategy to increase physical performance during cycling. Thus, carbohydrate mouth rinse protocols might be considered useful for training sessions performed with low carbohydrate/energy availability. Carbohydrate mouth rinse had the potential of increasing overall cycling power, and it was effective to increase cycling power during both climbing sections and flattish sections, ultimately reducing the total time to complete a 25.3 km-course. Nevertheless, the size of effect induced by the carbohydrate mouth rinse protocol on cycling performance was cataloged as small. However, the use of this protocol should only be recommended on an individual-basis, after experimenting with carbohydrate mouth rinsing while training, and in time trials that favors the use of this protocol.

## Ethics Statement

One week before the onset of the study, the participants were fully informed of the experimental procedures and the risks and discomforts associated with the research and gave their informed written consent to participate in the investigation. The study was approved by the Camilo José; Cela University Research Ethics Committee and has been performed in accordance with the ethical standards as laid down in the 1964 Declaration of Helsinki and its later amendments or comparable ethical standards.

## Author Contributions

GB-M and JDC conceived and designed the investigation, analyzed and interpreted the data, drafted the paper, and approved the final version submitted for publication.

### Conflict of Interest Statement

The authors declare that the research was conducted in the absence of any commercial or financial relationships that could be construed as a potential conflict of interest.
